# Raising alarm bells for a struggling sector: taking a new approach to improve the wellbeing of climate change professionals

**DOI:** 10.3389/fpsyg.2024.1404252

**Published:** 2024-07-19

**Authors:** Katharine Steentjes, Erin Roberts

**Affiliations:** ^1^School of Psychology, Faculty of Medicine, Health and Life Sciences, Swansea University, Swansea, United Kingdom; ^2^Centre for Climate Change and Social Transformations (CAST), Department of Psychology, University of Bath, Bath, United Kingdom; ^3^School of Biosciences, Geography and Physics, Faculty of Science and Engineering, Swansea University, Swansea, United Kingdom

**Keywords:** climate change, wellbeing, climate anxiety, burnout, mental health, community, creative methods, emotions

## Introduction

“Infuriated, hopeless and scared” are words recently used to describe how interviewed climate change experts feel about the current global crisis (Carrington, 2024).

Climate change is the greatest challenge facing the world today and we urgently need to find ways to transform our existing societies and lifestyles. However, we do need to find ways to realize this social transformation whilst upholding healthy standards of wellbeing and resilience. Public concern about climate change and its impacts has remained high despite other global events such as the COVID-19 pandemic, geopolitical turmoil and conflicts, extreme weather, and cost of living crises adding severe stressors in recent years (Demski et al., 2022). In fact, recent evidence seems to suggest that countries that have been experiencing conflicts and severe weather events tend to be the ones with higher levels of climate change worry (Steentjes et al., 2021; Poushter et al., 2022). Further research has demonstrated that concerns for the future, especially relating to the impacts of unaddressed climate change, have amplified into more severe negative emotional responses and mental health issues for many (Lawrance et al., 2021; Ogunbode et al., 2021; Pihkala, 2022). This opinion piece aims to raise awareness of the need to support the groups who are most vulnerable to the emotions of climate anxiety felt by many. In particular, we want to shed light on a group which has a crucial part to play in exploring and implementing potential ways to address the global crisis: climate change professionals. In addition, we offer thoughts on how the sector can start to address the mental health threat whilst at the same time creating opportunities to revive the sector's creativity and engagement.

Evidence of what is widely labeled as *climate anxiety*—a multifaceted concept that is closely interwoven with a range of other emotions such as worry, grief, and despair (Pihkala, 2020)—has started to appear more regularly among various groups worldwide, especially amongst younger generations (Hickman et al., 2021; Poortinga et al., 2023), indigenous peoples, and low income groups who are at increased risk of losing their homes, livelihoods, and cultures (Clayton et al., 2017). Along with these, climate change professionals—described as people that work, whether paid or unpaid, in roles such as climate science and related disciplines (including social sciences), charities, communicators, and policy advisors—are particularly susceptible to climate anxiety and distress due to their personal and occupational engagement with the topic (Clayton, 2018, 2020; Calabria et al., 2023). Current evidence on climate anxiety and the mental wellbeing of climate professionals is very scarce. Indeed, a recent study conducted in the US, provided, for the first time, evidence on the emotional state of climate change professionals specifically (Climate Burnout Report, 2023). Using a mixed-methods approach, the research set out to understand levels of climate burnout and the ability to regulate emotional reactions amongst professionals working in the climate change and environmental sector. Results identified very high levels of overall burnout, as measured by a short version of the Burnout Assessment Tool (Schaufeli et al., 2020; HadŽibajramović et al., 2022). Likewise, results from interviews and a survey amongst IPCC authors has revealed that 61% of respondents experienced grief, anxiety or other forms of distress frequently or infrequently (Tollefson, 2021). Some professionals even consider leaving the climate change sector due to frustrations caused by the barriers of effective climate action they experience (Latter et al., 2024). These trends, along with the stagnation or even scaling back of political efforts to rapidly decarbonize society in many countries in the global north of late (Shock delay to net-zero pledges turns UK from climate leader to laggard, 2023) places further pressure on the mental health on professionals that engage with climate change on a daily basis, and whose work and enthusiasm to create societal change is desperately needed to deal with the current environmental crisis.

## Why climate change professionals are struggling

Whilst this forum precludes an in-depth discussion of the topic, we want to highlight two interrelated concepts that we think are of particular relevance to climate change professionals' emotional state; these are *guilt* and a *lack of efficacy*.

### Guilt

Guilt, like most emotions associated with climate anxiety can, under specific circumstances, encourage climate action (Mallett, 2012; Whitmarsh et al., 2022). However, the collective (rather than personal) guilt that climate change can trigger in members of high-carbon emitting societies often does not offer a clear path to re-align this behavior, given the societal and structural boundaries (Suresh and Walter, 2022; Yacek, 2022). As scientists, communicators, or even leaders of the climate movement, climate professionals are likely to feel an expectation to lead by example when it comes to their own lifestyle choices (Gunster et al., 2018). A good understanding of carbon footprints, combined with strong environmental values and personal convictions are likely to cause feelings of guilt about one's own participation in creating the problem. Guilt has been found to be one of a swathe of negative emotions that people feel in response to climate change (Pihkala, 2022) but there is a clear lack of research on guilt specifically amongst climate professionals. Some related evidence suggests that guilt is an emotion often reported by climate activists (Kleres and Wettergren, 2017). If, as we hypothesize, guilt is felt especially when engaging in private sphere activities for climate change professionals, then this can create a tension that intensifies the threat to wellbeing, and in turn increases the need for recreational outlets for people dealing with the climate catastrophe.

### Lack of efficacy

Ample research highlights how important it is for people's wellbeing to feel they are in control and that their actions matter (e.g. Shoji et al., 2016). Specifically, the ever-increasing discrepancy between the urgent need for climate action and the lack of drastic actions to match this urgency is likely to undermine feelings of efficacy for people who are pushing for decisive climate action (e.g. Shoji et al., 2016). Indeed, professionals who experienced climate burnout reported that a feeling of being unable to solve the climate crisis was a primary reason for their burnout (Climate Burnout Report, 2023). The sentiment of low efficacy seems to also stem from the challenges associated with working within sometimes restrictive social and organizational systems at various scales. For example, a recent study showed that two thirds of participating climate change academics were thinking of leaving the sector completely as they felt unsupported by their institutions or more senior colleagues (Latter et al., 2024).

## Opportunity for a new and improved wellbeing of the climate change sector

In what follows, we outline what we believe to be a first starting point to addressing what is rapidly becoming a mental health crisis that threatens the resilience of a sector that is crucial for the development of effective climate change strategies. In this we adopt a framework that is rooted in climate psychology and offers a multi-faceted approach to wellbeing (Isham et al., 2023). By broadening the concept of wellbeing to encompass individual, collective and planetary wellbeing the crisis in the sector can be transformed into an opportunity to revive not only personal motivations but also improve the solutions designed by the sector.

We believe that it is crucial to create spaces and opportunities within work practices, where people can come together to express their worries and thoughts about the environmental crisis. Climate change professionals who experienced burnout reported to have felt very lonely and isolated (Climate Burnout Report, 2023), which suggests a lack of perceived acknowledgment and support by their professional environment. Institutional or peer support for mental health issues would send a strong message to counteract this isolation. However, we suggest that these safe spaces offer not only reflection on individual emotions but also create a sense of connectedness and community (Calabria et al., 2023). A sense of community had been identified by Clayton (2018) as a potential source of resilience for climate professionals; a sentiment which we strongly support. The importance of creating social connections to improve wellbeing for individuals but also communities and the planet is gaining recognition in climate psychology (Wilkie et al., 2022; Calabria et al., 2023; Isham et al., 2023).

However, the focus on community and togetherness should not be limited to the sectors own community if following the holistic framework. Instead, a stronger focus on connecting with other communities is needed; a practice that is already adopted to improve climate change engagement with wider publics. The Climate Psychology Alliance for example, offers Climate Cafés; online group sessions which provide opportunities for people to come together to share their climate change emotions, without judgement or expectation (Climate Psychology Alliance, 2024). A similar concept, for public-facing events that aim to encourage climate change engagement was developed and put into practice on a project called Climate Conversations (Büchs et al., 2015; Randall and Brown, 2015). Whilst also designed to discuss actionable solutions to climate change, the workshop series has been found to help participants, particularly people involved in the climate change movement, with their complex climate change emotions (Büchs et al., 2015).

In addition, we think that this new focus on togetherness and wellbeing can also offer opportunities for creativity and widening of discourses. Storytelling, for example, is a powerful tool of engagement with difficult topics or complex emotions, and it has been used in the context of climate change with diverse audiences under consideration of the existing complexities (Dahlstrom, 2014). This longstanding power of storytelling has prompted theoretical arguments for action-focused climate stories (De Meyer et al., 2021) to overcome climate fatalism and anxiety. One project exploring the impacts of storytelling workshops with climate professionals and school children provided positive results and demonstrated the multiple benefits of such an approach (Marks et al., 2023). Participating children reported that the storytelling helped them to engage with climate change in a creative, positive and hopeful way which encouraged them to express their emotions as well as motivate them to engage in climate action.

As demonstrated through the above-mentioned examples, the creation of safe spaces to encourage creative community engagement with climate change is closely connected to exploring meaningful action in response to this global crisis. It appears that people with climate anxiety seek validation and meaningful ways to channel their emotions. Ample research has explored the relationships between climate change emotions, behaviors and wellbeing with nuanced results trying to understand the causality of their connectedness (Sweeny and Dooley, 2017; Bouman et al., 2020; Stanley et al., 2021). What is clear is that pro-environmental behaviors are positively related to a person's subjective wellbeing (Zawadzki et al., 2020). If responded to with the right tools, then feelings of anxiety and dispair can be a motivational driver for action, whilst a failure of identyfing meaningful action worsens the menthal health crisis as well as blocks the actionalble societal shift needed (Ojala, 2023; Schwartz et al., 2023). A focus on developing and testing these tools can be just the what is needed to kickstart widespread action; action that can ensure public wellbeing and that of our planet (What happens when climate change and the mental-health crisis collide?, 2024). We suggest, that exploring how the climate change sector can create multi-purpose spaces with fellow climate professionals and other groups, is an opportunity to not only fight the mental wellbeing threat to the sector but also vitalize the solutions and thinking it produces.

## Discussion

This opinion piece has two key aims: firstly, to raise awareness of, and explore evidence around, the risk of growing climate anxiety and burnout amongst climate professionals, and secondly suggest how this crisis can be turned into an opportunity to revive, diversify and improve the sector's work. In times of increasing urgency of climate action and limited political will for drastic action, we need competent, healthy and resilient professionals who help to navigate the complexities of potential strategies. However, the limited evidence shows that many professionals experience climate anxiety and burnout (Tollefson, 2021; Climate Burnout Report, 2023). A trend we believe will continue if not addressed effectively.

Taking it a step further, we also suggest that opening these safe spaces to non-sectorial audiences, such as other struggling communities, offer the additional benefits of allowing more creativity and wider viewpoints into the climate change profession, as well taking on an authentic leadership role in healthy and effective engagement with climate change. This would improve the sector's resilience and help to retain some of its most passionate and impactful voices, whilst also (re)vitalizing collective action by creating further opportunities for creative thinking and problem-solving. We believe that in order to develop the creative, inclusive, just and drastic solutions required to address the climate catastrophe, we need to admit to the mental health crisis and take a holistic approach when addressing this through the inclusion of community, nature and creative methods.

By no means do we suggest a comparison of the mental wellbeing risks for climate change professional with those felt by vulnerable groups, such as indigenous peoples, minority groups, and people already losing their homes and/or livelihoods to climate change. We do feel, however, that it is vital to take a closer look at the wellbeing of the sector to improve their discourse, connectedness and creativity. This, we hope, will also diversify the sector itself and the solutions discussed.

## Author contributions

KS: Writing – original draft, Writing – review & editing, Conceptualization. ER: Writing – original draft, Writing – review & editing.

## References

[B1] Bouman T. Verschoor M. Albers C. J. Böhm G. Fisher S. D. Poortinga W.. (2020). When worry about climate change leads to climate action: how values, worry and personal responsibility relate to various climate actions. Glob. Environ. Change 62:102061. 10.1016/j.gloenvcha.2020.102061

[B2] Büchs M. Hinton E. Smith G. (2015). “It helped me sort of face the end of the world”: the role of emotions for third sector climate change engagement initiatives. Environ. Values 24, 621–640. 10.3197/096327115X14384223590177

[B3] Calabria L. Peters C. Williams M. (2023). A compassion focused approach to understanding the mental health of climate scientists. The Cognitive Behaviour Therapist. Cardiff: Cardiff University.

[B4] Carrington D. (2024). We asked 380 top climate scientist what they felt about the future: they are terrified but determined to keep fighting. The Guardian, 8 May. Availabe online at: https://www.theguardian.com/environment/ng-interactive/2024/may/08/hopeless-and-broken-why-the-worlds-top-climate-scientists-are-in-despair (accessed July 9, 2024).

[B5] Clayton S. (2018). Mental health risk and resilience among climate scientists. Nat. Clim. Change 8, 260–261. 10.1038/s41558-018-0123-z38484753

[B6] Clayton S. (2020). Climate anxiety: psychological responses to climate change. J. Anxiety Disord. 74:102263. 10.1016/j.janxdis.2020.102263 32623280

[B7] Clayton S. Manning C. M. Krygsman K. Speiser M. (2017). Mental Health and Our Changing Climate: Impacts, Implications, and Guidance. Washington, DC: American Psychological Association, and ecoAmerica.

[B8] Climate Burnout Report (2023). Climate Critical. Available online at: https://www.climatecritical.earth/report (accessed July 9, 2024).

[B9] Climate Psychology Alliance (2024). Find Support website. Available online t: https://www.climatepsychologyalliance.org/index.php/find-support (accessed March 13, 2024).

[B10] Dahlstrom M. F. (2014). Using narratives and storytelling to communicate science with nonexpert audiences. Proc. Natl. Acad. Sci. 111(supplement_4), 13614–13620. 10.1073/pnas.1320645111 25225368 PMC4183170

[B11] De Meyer K. Coren E. McCaffrey M. Slean C. (2021). Transforming the stories we tell about climate change: from ‘issue' to ‘action'. Environ. Res. Lett. 16:015002. 10.1088/1748-9326/abcd5a

[B12] Demski C. Steentjes K. Poortinga W. (2022). Public worry about climate change and energy security in the cost-of-living crisis. CAST Briefing 17. The Centre for Climate Change and Social Transformations, Bath.

[B13] Gunster S. Fleet D. Paterson M. Saurette P. (2018). “Why don't you act like you believe it?”: competing visions of climate hypocrisy. Front. Commun. 3:49. 10.3389/fcomm.2018.00049

[B14] HadŽibajramović E. Schaufeli W. De Witte H. (2022). Shortening of the burnout assessment tool (BAT)—from 23 to 12 items using content and Rasch analysis. BMC Public Health 22:560. 10.1186/s12889-022-12946-y 35313849 PMC8939057

[B15] Hickman C. Marks E. Pihkala P. Clayton S. Lewandowski R. E. Mayall E. E.. (2021). Climate anxiety in children and young people and their beliefs about government responses to climate change: a global survey. Lancet Planet. Health 5, e863–e873. 10.1016/S2542-5196(21)00278-3 34895496

[B16] Isham A. Morgan G. Kemp A. H. (2023). Nurturing wellbeing amidst the climate crisis: on the need for a focus on wellbeing in the field of climate psychology. Front. Psychol. 14:1205991. 10.3389/fpsyg.2023.1205991 37575429 PMC10413562

[B17] Kleres J. Wettergren Å. (2017). Fear, hope, anger, and guilt in climate activism. Soc. Mov. Stud. 16, 507–519. 10.1080/14742837.2017.134454637610987

[B18] Latter B. Demski C. Capstick S. (2024). Wanting to be part of change but feeling overworked and disempowered: researchers' perceptions of climate action in UK universities. PLOS Clim. 3:e0000322. 10.1371/journal.pclm.0000322

[B19] Lawrance E. Thompson R. Fontana G. Jennings N. (2021). “The impact of climate change on mental health and emotional wellbeing: current evidence and implications for policy and practice,” in Grantham Institute briefing paper, 36. Imperial College London.36165756

[B20] Mallett R. K. (2012). Eco-guilt motivates eco-friendly behavior. Ecopsychology 4, 223–231. 10.1089/eco.2012.0031

[B21] Marks E. Atkins E. Garrett J. K. Abrams J. F. Shackleton D. Hennessy L.. (2023). Stories of hope created together: a pilot, school-based workshop for sharing eco-emotions and creating an actively hopeful vision of the future. Front. Psychol. 13:8402. 10.3389/fpsyg.2022.1076322 36687969 PMC9853550

[B22] Ogunbode C. A. Pallesen S. Böhm G. Doran R. Bhullar N. Aquino S.. (2021). Negative emotions about climate change are related to insomnia symptoms and mental health: cross-sectional evidence from 25 countries. Curr. Psychol. 42, 845–854. 10.1007/s12144-021-01385-4

[B23] Ojala M. (2023). Hope and climate-change engagement from a psychological perspective. Curr. Opin. Psychol. 49:101514. 10.1016/j.copsyc.2022.101514 36502586

[B24] Pihkala P. (2020). Anxiety and the ecological crisis: an analysis of eco-anxiety and climate anxiety. Sustainability 12, 7836. 10.3390/su12197836

[B25] Pihkala P. (2022). Toward a taxonomy of climate emotions. Front. Clim. 3:738154. 10.3389/fclim.2021.738154

[B26] Poortinga W. Demski C. Steentjes K. (2023). Generational differences in climate-related beliefs, risk perceptions and emotions in the UK. Commun. Earth Environ. 4:229. 10.1038/s43247-023-00870-x37484093

[B27] Poushter J. Fagan M. Gubbala S. (2022). Climate *Change Remains Top Global Threat across* 19-*Country Survey*. Washington, DC: Pew Research Center.

[B28] Randall R. Brown A. (2015). In *Time* for *Tomorrow*. The Carbon Conversations Handbook. Washington, DC: Surefoot Effect, Community Interest Company.

[B29] Schaufeli W. B. Desart S. De Witte H. (2020). Burnout Assessment Tool (BAT)—development, validity, and reliability. Int. J. Environ. Res. Public Health 17:9495. 10.3390/ijerph17249495 33352940 PMC7766078

[B30] Schwartz S. E. Benoit L. Clayton S. Parnes M. F. Swenson L. Lowe S. R.. (2023). Climate change anxiety and mental health: environmental activism as buffer. Curr. Psychol. 42, 16708–16721. 10.1007/s12144-022-02735-6 35250241 PMC8883014

[B31] Shock delay to net-zero pledges turns UK from climate leader to laggard (2023). Nature 621, 657–658. 10.1038/d41586-023-02987-7 37749341

[B32] Shoji K. Cieslak R. Smoktunowicz E. Rogala A. Benight C. C. Luszczynska A.. (2016). Associations between job burnout and self-efficacy: a meta-analysis. Anxiety Stress Coping 29, 367–386. 10.1080/10615806.2015.1058369 26080024

[B33] Stanley S. K. Hogg T. L. Leviston Z. Walker I. (2021). From anger to action: differential impacts of eco-anxiety, eco-depression, and eco-anger on climate action and wellbeing. J. Clim. Change Health 1:100003. 10.1016/j.joclim.2021.100003

[B34] Steentjes K. Demski C. Poortinga W. (2021). Public perceptions of climate change and policy action in the UK, China, Sweden and Brazil (CAST Briefing Paper 10), Isslocue.

[B35] Suresh S. Walter N. (2022). Guilt by association, change by individuation: examining the role of guilt and efficacy in mitigating collective risks. J. Appl. Soc. Psychol. 52, 1049–1061. 10.1111/jasp.12911

[B36] Sweeny K. Dooley M. D. (2017). The surprising upsides of worry. Soc. Personal. Psychol. Compass 11:e12311. 10.1111/spc3.12311

[B37] Tollefson J. (2021). Top climate scientists are sceptical that nations will rein in global warming. Nature 599, 22–24. 10.1038/d41586-021-02990-w 34725476

[B38] What happens when climate change and the mental-health crisis collide? (2024). Nature 628:235. 10.1038/d41586-024-00993-x 38600269

[B39] Whitmarsh L. Player L. Jiongco A. James M. Williams M. Marks E.. (2022). Climate anxiety: what predicts it and how is it related to climate action? J. Environ. Psychol. 83:101866. 10.1016/j.jenvp.2022.10186637784133

[B40] Wilkie L. Fisher Z. Kemp A. H. (2022). The ‘rippling' waves of wellbeing: a mixed methods evaluation of a surf-therapy intervention on patients with acquired brain injury. Sustainability 14:9605. 10.3390/su14159605

[B41] Yacek D. W. (2022). “Anxiety, guilt and activism in teaching about climate change,” in Creating Green Citizens: Bildung, Demokratie und der Klimawandel, eds. J. Drerup, F. Felder, V. Magyar-Haas, and G. Schweiger (Berlin: Springer), 115–134. 10.1007/978-3-662-63376-2_7

[B42] Zawadzki S. J. Steg L. Bouman T. (2020). Meta-analytic evidence for a robust and positive association between individuals' pro-environmental behaviors and their subjective wellbeing. Environ. Res. Lett. 15:123007. 10.1088/1748-9326/abc4ae

